# Mr. Hugo's Case of Rupture of the Uterus

**Published:** 1808-03

**Authors:** Thomas Hugo

**Affiliations:** Creditou


### *To the Editors of the Medical and Physical Journal.*

GENTLEMEN,

I HAVE the pleasure of communicating to you, for insertion in the Medical and Physical Journal, the following Case of recovery after a Rupture of the Uterus.

---

Mrs. Skinner, the wife of a farmer in this neighbourhood, aged 39 years, is of a strong and healthy constitution, but was lately much weakened by a severe peripneumony. Previously to the confinement which has afforded the subject of this case, I have attended her in four labours. They were all difficult in consequence of the small dimensions of the pelvis, and in two of them, where the children were very large, and the bones of the cranium much ossified, I was obliged, on account of the dangerous state of the mother, to have recourse to the crotchet; as the most violent action of the uterus, for three and four days, was unable to bring the head so low into the pelvis as to admit the application of any other instrument,—and her recovery was each time very slow and difficult. In the two other labours, the children being small, and the cranium less ossified, the delivery was effected by the action of the uterus.

She was taken in labour for the fifth time, at the full period of gestation, early on the morning of September 30 last. On my arrival, I found the os uteri fully dilated, the external parts wholly relaxed, the head of the child resting on the pubes, the membranes entire, and the pains frequent and very strong, accompanied with a small discharge of blood. During one of those pains, the membranes were protruded through the os externum, and in that state the waters were discharged. The head now gradually advanced, and, in about three hours, became forced into the narrow part of the pelvis. When it had arrived in this situation, though, during every pain, it still appeared slowly to descend, she complained of a more continued and severe pain in the lower part of the uterus, with so great a degree of restlessness, that she could scarcely be prevailed on to remain in bed. As her disposition is naturally passive and accommodating, I particularly noticed this circumstance; and for the purpose of moderating the action of the uterus, I administered, without delay, a full dose of tinct. opii. The pains, within a quarter of an hour after this, this, had *entirely* ceased, which I then attributed to the effect of the opiate: still, however, the restlessness was not lessened, and it was accompanied with great anxiety.

She soon afterwards called me to her bed-side, and requested me to apply my hand on the abdomen; as she expressed herself, that during the *last pain*, "something had slipped out of its place." I perceived an irregular tumour, about the size of a man's fist on the lower part of the abdomen towards the right side. I could not distinctly feel that it contained any *particular* part of the child; but the circumstance, connected with the other symptoms, impressed me with the belief, that the uterus had ruptured at this part. The light of the morning enabled me also to observe her countenance. She was pale, breathed laboriously, and was apparently sinking. A violent vomiting supervened, the pulse was very quick and feeble, and the extremities covered with cold perspiration.

As I considered that nothing but speedy delivery could afford her the smallest chance of recovery, from a situation which was almost hopeless, I proceeded, as soon as I could obtain the proper instruments, to open the cranium, which still preserved its situation in the pelvis. But on the application of the perforator, before I could use a sufficient degree of force even to penetrate the scalp, it suddenly receded into the cavity of the uterus. I immediately followed it with my hand. The uterus was so relaxed and yielding, that I was enabled, with the greatest ease, to bring down both feet into the vagina.

It was not to be expected that the child could be extracted through a narrow pelvis in so short a time as to preserve its life, and, indeed, it was this consideration, which at first determined me on the use of the *crotchet*, in preference to turning; the application of any other instrument being, from the situation of the head, impracticable. The child proved to be so large, that it was with great difficulty I could bring the *thorax* through the pelvis, and it was not till I had applied the blunt hook over one of the shoulders, that I could bring the body so low as to reach the arms: but I was unable to deliver the head, till I had lessened its bulk, and assisted by fixing the crotchet on the inside. As it was in vain to wait for the natural expulsion of the placenta, I soon after the delivery, proceeded to introduce my hand again into the uterus.

If any doubt had before remained of the nature of this case, it was now wholly removed, as I passed my hand without difficulty, through the aperture into the abdomen.

I applied

I applied my fingers on the smooth *internal* surface of that cavity, when the gentleman who assisted, and myself, were both satisfied, by placing our hands, at the same time, on the *outside*, that nothing but the thin parietes of the abdomen intervened. The ruptured part, as nearly as I could ascertain, was in the anterior part of the cervix uteri, extending also into the vagina. There was fortunately none of the intestines in the way, nor did I think myself justified in searching for them, but hastened to withdraw my hand into the uterus, when I removed the placenta, and resigned, as I had then too much reason to fear, my unhappy patient to her fate.

No extraordinary discharge followed the delivery. She continued during the remaining part of this day in a very languid state, with violent and frequent vomiting.—On the following morning, having had some sleep during the night, she appeared considerably revived. The pulse was 120 in a minute, and stronger. There was no general enlargement, nor pain in the abdomen, except on pressure, when the uterus was described to be exquisitely tender, and was felt to be contracted about the size of a child's head. She had voided urine without difficulty, but there had been very little discharge of lochia, and scarcely any thing had remained on the stomach. In this state, she continued with little variation for the first four days.—The regimen during this time was strictly antiphlogistic, and the medicines were saline draughts, with gentle aperients, and an opiate at night.

From the 5th to the 12th day, she became gradually into a state of the most distressing restlessness and anxiety: constantly endeavouring, but unable, to change her posture. She repeatedly vomited a *dark coloured* fluid, and had frequent motions of a similar appearance. The pulse was very irritable and weak, and varying from 130 to 150 in a minute; the abdomen was enlarged and painful; the uterus extremely tender; the lochia serous, and in very small quantity.—The Peruvian bark in different preparations was now directed to be taken freely, and light nutriment, in all the forms that could be devised, was prescribed for her. But almost every thing was ejected from the stomach, and except the opiate at night, she could not, at last, be prevailed on to take any medicine, and with great difficulty, food. A few hours sleep, procured by the opiate, was the only relief she experienced.

Such was her situation on the 12th day, at which time the size and tension of the abdomen indicated an extravasation sation of fluid into that cavity. As no medicine could be retained on the stomach, the ungt. hydrargyri fort. was directed to be rubbed into the abdomen twice a day, with a view, if possible, to produce its absorption.

On the following morning, the enlargement was much subsided, in consequence of her having had several *very profuse* motions during the night. The uterus was become less tender; the pulse reduced to 100, and the restlessness, anxiety, and every other unfavourable symptom, abated. From this day, she continued gradually to amend, but whether this abatement of the unfavourable symptoms was occasioned by the mercurial action, or whether the application had any effect in producing the extraordinary discharge from the bowels, is uncertain. There appears, however, to have been scarcely time, nor does it seem that the quantity used was sufficient for so powerful an operation.—Yet though I was more disposed to attribute it to a critical effort of nature, than to an effect of the medicine employed, I considered it, at least, as an inducement to continue its use: and the amendment taking place so soon after its first application, my patient was very solicitous to persevere in it. In eight or ten days, a soreness of the gums obliged me to desist. During this period, a larger discharge of lochia came on; the stomach by degrees retained food, and the more alarming symptoms had so far subsided, that I could not but indulge the most pleasing expectation of her recovery.

About three weeks after delivery, she was attacked by the peculiar swelling of the leg and thigh incident to lying-in women, (Phlegmatia Dolens.) This considerably retarded the progress of her recovery, and did not wholly subside for another fortnight.

The debility occasioned by so long and painful a confinement, may be supposed to have been very great, and it was not till five weeks after delivery there could be said to be any return of strength. She was enabled then to be taken out of bed for an hour daily, and her appetite began to increase. The swelling of the abdomen was entirely reduced, but there was still felt, on pressure, a tenderness in the region of the uterus. She also complained of a very troublesome sensation near the spine of the left ilium, when in an erect posture, or when lying on the opposite side, which she described "as if something was drawing away from that part: probably, the consequence of an adhesion formed there, during the period of peritoneal inflammation. She went on rapidly, from this time, in a state state of convalescence, and is now so well recovered, as, except a diminution of strength, to feel little inconvenience from the accident.

The above is an addition to the few instances of recovery, with which we are acquainted, from the most formidable accident that can occur in the practice of midwifery; and though there may be but little practical information to be derived from it, the recording of it must tend to inspire us with a greater degree of confidence in the powers of nature, even in the most desperate cases, where the impediments to the exertion of those powers are removed.— The only two instances of ruptured uterus which have fallen under my own observation have been in women with narrow pelves, and in both of them it took place in or near the cervix uteri. A degree of weakness in any part of that organ, disproportioned to the resistance to be overcome, must always predispose it to rupture. But if the weakness of structure should exist principally near its *cervix,* that part, *after the evacuation of the waters,* is in danger of becoming violently compressed between the head of the child and the edge or some projecting part of the pelvis; and in such a situation, the probability of its giving way must be very great. Whenever, therefore, delivery is protracted on account of an extraordinary degree of resistance, either from the smallness of the pelvis, or a rigidity of the soft parts, the practitioner has been much cautioned to avoid the premature rupturing of the membranes. And if, *after that has taken place,* the woman should complain of an unusually severe pain, confined to a particular *part* of the uterus, distinct from, more agonizing, and sometimes more constant than the labour pains, symptoms which indicate the too forcible compression of some *portion* of the uterus, he cannot be too much upon his guard, nor too prompt in endeavouring to moderate its action, and to lessen the resistance it has to overcome.

Both these purposes, if the difficulty should depend solely on a *rigidity of the parts to be dilated,* may, as far as it is in our power, be accomplished by large doses of opium, with blood-letting in proportion to the strength of the patient. But, as is more frequently the case, if the resistance should be occasioned by the *narrowness of the pelvis,* it becomes a subject of consideration, whether the magnitude of the danger we apprehend and wish to avert, will not justify us in effecting the delivery without further delay:—by turning, if it be thought compatible with the safety of the mother and the child, or otherwise even by lessening the bulk of the head, should it be situated too high to admit of our applying the forceps or the vectis.

When, notwithstanding all our precautions, a rupture of the uterus has unhappily taken place, the necessity of a speedy delivery is, I believe, generally considered as unquestionable.—Every additional case of recovery, under these circumstances, should impress us the more strongly with the propriety of the practice, and deter us, under an idea of the unavoidable fatality of the accident, as has been sometimes the case, from allowing the woman to remain undelivered, which can afford the least possible chance to the mother, and must *necessarily* prove the destruction of the child.

I am, &c.

THOMAS HUGO.

*Crediton, December* 24, 1807.

---

